# The Novel Wheat Transcription Factor TaNAC47 Enhances Multiple Abiotic Stress Tolerances in Transgenic Plants

**DOI:** 10.3389/fpls.2015.01174

**Published:** 2016-01-18

**Authors:** Lina Zhang, Lichao Zhang, Chuan Xia, Guangyao Zhao, Jizeng Jia, Xiuying Kong

**Affiliations:** Key Laboratory of Crop Gene Resources and Germplasm Enhancement, Ministry of Agriculture, The National Key Facility for Crop Gene Resources and Genetic Improvement, Institute of Crop Science, Chinese Academy of Agricultural SciencesBeijing, China

**Keywords:** NAC transcription factor, abiotic stress, ABRE, wheat, ABA

## Abstract

NAC transcription factors play diverse roles in plant development and responses to abiotic stresses. However, the biological roles of NAC family members in wheat are not well understood. Here, we reported the isolation and functional characterization of a novel wheat *TaNAC47* gene. *TaNAC47* encoded protein, localizing in the nucleus, is able to bind to the ABRE *cis*-element and transactivate transcription in yeast, suggesting that it likely functions as a transcriptional activator. We also showed that *TaNAC47* is differentially expressed in different tissues, and its expression was induced by the stress treatments of salt, cold, polyethylene glycol and exogenous abscisic acid. Furthermore, overexpression of *TaNAC47* in *Arabidopsis* resulted in ABA hypersensitivity and enhancing tolerance of transgenic plants to drought, salt, and freezing stresses. Strikingly, overexpression of *TaNAC47* was found to activate the expression of downstream genes and change several physiological indices that may enable transgenic plants to overcome unfavorable environments. Taken together, these results uncovered an important role of wheat *TaNAC47* gene in response to ABA and abiotic stresses.

## Introduction

Abiotic stress is one of the main factors influencing growth, development and yield of plants worldwide. The exploitation and utilization of stress-tolerant plants will become more significant in the saline-alkali land and water shortage areas. Recently, progress has been made in identifying beneficial stress-related genes that can enhance the tolerance of plants to abiotic stresses (Tran et al., 2010; Nakashima et al., 2012). Transcription factors (TFs) are pivotal regulators involving in the response to abiotic stress, and overexpression of TF genes commonly improved a plant’s tolerance to abiotic stress. The NAC protein forms one of the largest families of plant-specific TFs (Olsen et al., 2005). They were derived from three genes containing particular domains of NAM (no apical meristem), ATAF (*Arabidopsis* transcription activation factor) and CUC (cup-shaped cotyledon) (Souer et al., 1996; Aida et al., 1997). Typically, a NAC transcription factor harbors a highly conserved N-terminal NAC domain and a variable C-terminal transcription regulatory (TR) region (Ernst et al., 2004; Olsen et al., 2005). The nearly invariable N-terminal NAC domain is responsible for nuclear localization, DNA binding, and formation of homodimers or heterodimers. This domain contains approximately 150 amino acids and is classified as an A-E subdomain (Olsen et al., 2005). In contrast, the C-terminal region is diverse and can function as a transcriptional activator or repressor (Delessert et al., 2005; Kim et al., 2007; Yamaguchi et al., 2010; Puranik et al., 2011).

Numerous reports have demonstrated that NAC TFs are involved in a number of biological processes, such as controlling cell division by mediating cytokinin signaling (Kim et al., 2006), regulating the growth of plant cells (Kato et al., 2010), lateral root development (He et al., 2005; Quach et al., 2014), and leaf senescence (Guo and Gan, 2006; Yang et al., 2011; Shah et al., 2013); inducing phytoalexin biosynthesis (Saga et al., 2012), formation of secondary walls (Mitsuda et al., 2007; Zhao et al., 2010), flower formation (Hendelman et al., 2013) and responding to pathogen infection (Voitsik et al., 2013; Yokotani et al., 2014), seed development (Park et al., 2011) and fiber development (Zhao et al., 2014). Additionally, many members of the NAC TF family can coordinate the response to abiotic stress. In rice, expressions of *OsNAC5*, *OsNAC6*, *OsNAP*, and *SNAC1* were induced by drought, cold and high salinity. Overexpression of these four genes in transgenic rice improved the tolerance to high salinity and dehydration. Wheat plants expressing the *SNAC1* gene exhibited increased tolerance to drought and salinity (Nakashima et al., 2007; Takasaki et al., 2010; Saad et al., 2013; Chen et al., 2014). Similarly, transgenic rice overexpressing *OsNAC045* exhibited enhanced resistance to both drought and salt stresses (Zheng et al., 2009). Root-specific overexpression of the *OsNAC9* and *OsNAC10* genes resulted in enlarged roots and enhanced the drought tolerance of transgenic rice that led to significantly increasing the grain yield under field drought conditions (Jeong et al., 2010; Redillas et al., 2012). Recently, the characterization of the roles of stress-related NAC transcription factors has been reported in wheat. Two genes, *TaNAC4* and *TaNAC8*, were involved in stripe rust pathogen infection and abiotic stresses (Xia et al., 2010a,b). *TaNAC69* expression was up-regulated by multiple abiotic stresses in wheat, and overexpression of *TaNAC69* in transgenic wheat enhanced the expression levels of stress up-regulated genes and dehydration tolerance (Xue et al., 2011). Moreover, overexpression of *TaNAC2*, *TaNAC2a* and *TaNAC67* improves the tolerance of transgenic plants to abiotic stresses (Tang et al., 2012; Mao et al., 2012, 2014).

To interpret the possible molecular regulatory mechanisms underlying the plant response to abiotic stress and accelerate the use of the *NAC* gene to facilitate engineering transgenic wheat, we characterized an abiotic stress-related gene, *TaNAC47*, from a full-length wheat cDNA library. Its expression profiles in response to polyethylene glycol (PEG), salt, cold, and exogenous ABA treatments were examined in wheat by using the quantitative real-time PCR (RT-qPCR) approach. Its function in abiotic stresses tolerance was evaluated by ectopic expression of *TaNAC47* in *Arabidopsis*. There were no obvious morphological differences between the transgenic and WT *Arabidopsis* plants under normal growth conditions. Together, results collected in this study indicated that *TaNAC47* is likely a candidate gene that will be useful for improving stress tolerance.

## Materials and Methods

### Plant Materials and Abiotic Stress Treatments

For expression analysis of *TaNAC47*, young spikes, leaves, stems and roots were sampled from Chinese Spring (CS) wheat. The wheat cv. Hanxuan 10 (drought resistant), Chadianhong (salt resistant) and CS were subjected to drought, salt, low temperature and exogenous ABA treatments. Wheat seeds were germinated and grown with distilled water at 25°C under a 16 h light/8 h dark cycle. Ten-day-old seedlings were treated with 16.1% PEG-6000 (-0.5 MPa), 250 mM NaCl, and 200 μM ABA at 4°C for 0, 1, 3, 6, 12, 24, and 48 h. All of the treated samples were immediately frozen with liquid nitrogen and stored at -80°C for RNA isolation. *Arabidopsis thaliana* Columbia-0 was used for transgenic study of *TaNAC47*. CS wheat was used to analyze the genomic sequence of the gene.

### Genomic Sequence Isolation and Analysis of *TaNAC47*

The *TaNAC47* cDNA sequence was obtained by sequencing the cDNA plasmid libraries in our laboratory. Gene-specific primers (forward 5′- CCAATGAAGATGAACCCC -3′ and reverse 5′-AATGCTACTGTGAGAGAG-3′) were designed to perform genomic sequencing in CS wheat. The PCR products were cloned into the vector of pEASY-T1 and sequenced. The sequence alignment between the cDNA and the genomic DNA of the gene was used to analyze the exons and introns of the genomic DNA. The theoretical molecular weight and isoelectric point were calculated using ExPASy^1^. The NAC domain region was identified with SCANPROSITE^2^.

### Cloning of the Promoter Sequences and Analysis of *Cis*-acting Element

The promoter sequence of *TaNAC47* was obtained by using *TaNAC47* genomic DNA as the query sequences to blast *Ae. tauschii* genome sequence database (Jia et al., 2013). The 1,500 bp of sequence upstream from the initiation codon (ATG) of *TaNAC47* was used to analyze the *cis*-acting elements by using the PLACE database^3^.

### Subcellular Localization of the TaNAC47 Protein

The full-length coding sequence of *TaNAC47* was fused to the modified pEarleyGate-GFP vector under the control of the cauliflower mosaic virus (CaMV) 35S promoter to generate the 35S::GFP-TaNAC47 fusion construct by the gateway method. The 35S::GFP-TaNAC47 fusion protein and 35S::GFP alone were introduced into epidermal cells of *N. benthamiana* separately *via* an *Agrobacterium*-mediated method (Sheludko et al., 2007). The transformed *N. benthamiana* leaves were grown under the normal conditions for 2-6 days. The signals were observed and photographed using confocal laser scanning microscopy (Zeiss Lsm 700, Zeiss, Jena, Germany).

### Transcription Activation Assay in Yeast

To investigate the transcriptional activity of TaNAC47 protein, the full-length coding sequence of *TaNAC47* was amplified using a pair of gene-specific primers containing the attB sites (forward primer 5′ -GGGGACAAGTTTGTACAAAAAAGCAGGCTTAATGGTGATGGCGGCGGCG-3′ and reverse primer 5′-GGGG ACCACTTTGTACAAGAAAGCTGGGTTCAGAAGAAGAATGGGCTGA-3′; attB sites underlined) and then fused with the DNA-binding domain (BD) in a pDEST32 vector. The fused construct *pDEST32-TaNAC47*, *pGAL4* and the *pDEST32* vector were transformed into the yeast strain AH109 separately. The transformants were plated on the medium without histidine, leucine, and adenine for the selection of transactivation properties of the reporter constructs.

### Yeast One-Hybrid Assays

Full-length *TaNAC47* gene was fused in frame with the GAL4 DNA-activation domain (AD) in a vector pDEST22. The bait construct containing the hexamer ABRE/mABRE sequence was provided by Professor Jun Zhao (Biotechnology Research Institute, Chinese Academy of Agricultural Sciences, Beijing, China). The recombinant plasmid *pDEST22*-*TaNAC47* and the ABRE bait plasmid were co-transformed into the yeast strain YM4271 using the Frozen-EZ yeast transformation method (Zymo Research, Irvine, CA, USA). Two different combinations, (i) *GAL4-AD-TaNAC47* and mutated forms of ABRE sequence (mABRE); (ii) the *GAL4-AD* and ABRE sequence were developed, along with *GAL4-AD* and mABRE used as control were transformed into the yeast strain YM4271 separately. The yeast transformants were cultured on medium of SD/Leu^-^ Trp^-^ at 30°C for about 2 days, and the colonies were then dropped onto the medium of SD/Leu^-^ Trp^-^ His^-^ containing 0.5 mM 3-AT.

### Quantitative RT-PCR Analysis

Total RNA extraction and reverse transcription were performed using the TRIZOL reagent and SuperScript^TM^II reverse transcriptase (Invitrogen, Carlsbad, CA, USA). The RT-qPCR reaction mix included 5 μL of SYBR Premix EX Taq^TM^ (Takara, Shiga, Japan), 2 μL of 2 μM of each primer, 2 μL of cDNA and distilled water to a final volume of 20 μL, and amplified for 40 cycles with each cycle consisting of 20 s at 95°C; 20 s at 55°C; and 30 s at 72°C on the Applied Biosystems 7500 real time RT-qPCR instrument (Applied Biosystems, Foster City, CA, USA). Wheat *Tubulin* gene (NCBI accession No. AF251217.1) and *Arabidopsis Atactin* gene (NCBI accession No. NM_112764) were used as the internal reference, and the 2^-ΔΔCT^ method was used to perform the quantitative analysis. All RT-qPCR reactions data were obtained from three independent experiments. The primers used for the RT-qPCR were listed in Supplementary Table S2.

### Generation of *TaNAC47* Transgenic *Arabidopsis* Plants

The full-length coding sequence of *TaNAC47* was introduced into the pEarleyGate 100 vector to generate the 35S::*TaNAC47* construct. The construct was transformed into the *Agrobacterium tumefaciens* strain GV3101 (90RK), and then delivered into *A. thaliana* ecotype Col-0 through the floral dip method (Clough and Bent, 1998). The positive transgenic plants were screened by spraying a 0.002% (v/v) Basta solution and then confirmed by RT-PCR. T_3_ lines exhibiting 100% Basta resistance were considered to be homozygous and selected for further experiments.

### Analysis of Abiotic Stress Tolerance and ABA Sensitivity in Transgenic *Arabidopsis* Plants

For determining the salt tolerance in transgenic plants, five-day-old seedlings grown vertically on MS plates were moved to new plates with addition of different concentrations of NaCl (0, 150, and 200 mM) and continued for 5 days. The root lengths of the seedlings were recorded.

For freezing tolerance test, 2-week-old transgenic and WT *Arabidopsis* seedlings were grown under a normal condition and then subjected to -10°C cold treatment for 3 h. Seedlings were then placed at 5°C for 2 h before transferring to a normal condition at 22°C.

To study drought tolerance of transgenic *Arabidopsis*, ten seedlings were grown in rectangular plates (4 cm deep) with a mixture of vermiculite and humus, and kept well-watered. The seedlings were then cultivated in a phytotron chamber without watering for about 35 days, followed by rehydrating the seedlings for 5 days. The survival rates of transgenic and WT seedlings were statistical analyzed.

For analyzing the sensitivity of transgenic seedlings to ABA, transgenic and WT seedlings grown on the MS plates for 5 days were transferred to new MS plates containing different concentrations of ABA and continued for 7 days. The root lengths of the seedlings were measured. All of the experiments described above were repeated three times.

### Measurements of Relative Electrolyte Leakage, Soluble Sugar, Proline Content, and Water Loss Rate

For determining the water loss rate, leaves were harvested from 3-week-old seedlings of transgenic and WT plants and weighed immediately. The leaves were then placed on the lab bench (20-22°C, humidity 45-60%) and weighed at designated time points. The water loss rate was calculated related to the initial fresh weight of the samples. Each measurement was performed in triplicate.

The free proline concentrations were measured according to previously described method (Zhang et al., 2012). The electrolyte leakage and soluble sugar content were evaluated following the method described previously (Lehner et al., 2006; Cao et al., 2007).

All the measurements were performed with ten plants in triplicate, and Student’s *t*-test was performed for statistical analysis.

## Results

### Sequence Characterization of the *TaNAC47* Gene

The full-length *TaNAC47* cDNA was 1,277 bp and was predicted to encode a 271-amino acid protein harboring a molecular weight of 32.37 kD with a *pI* of 5.13. The deduced TaNAC47 protein contained a conserved NAC domain (19-169 aa). The *TaNAC47* gene sequence was submitted to GenBank with the accession number KT345698. Results from blast analysis revealed that TaNAC47 shared 87.6% identity to another wheat abiotic stressed responsive NAC protein TaNAC67.

To analyze the gene structure of *TaNAC47*, the genomic sequence was cloned from the hexaploid CS wheat. A comparison of the genomic sequence and the corresponding cDNA sequence of *TaNAC47* revealed that the *TaNAC47* gene did not possess any intron.

Analysis of *cis*-acting regulatory element in the promoter region of *TaNAC47* revealed the presence of basic components and stress-responsive element-binding motifs, including A-boxes, C-boxes, CAAT-boxes, GATA-boxes, and the abiotic stress response *cis*-elements (ABRE, DRE/CRT, HSE, and LTRE; Supplementary Table S1). Collectively, the presence of these *cis*-elements suggested that the *TaNAC47* gene may play a role in response to abiotic stresses, most likely via an ABA-dependent pathway.

### Expression Pattern of *TaNAC47* in Wheat

**FIGURE 1:**
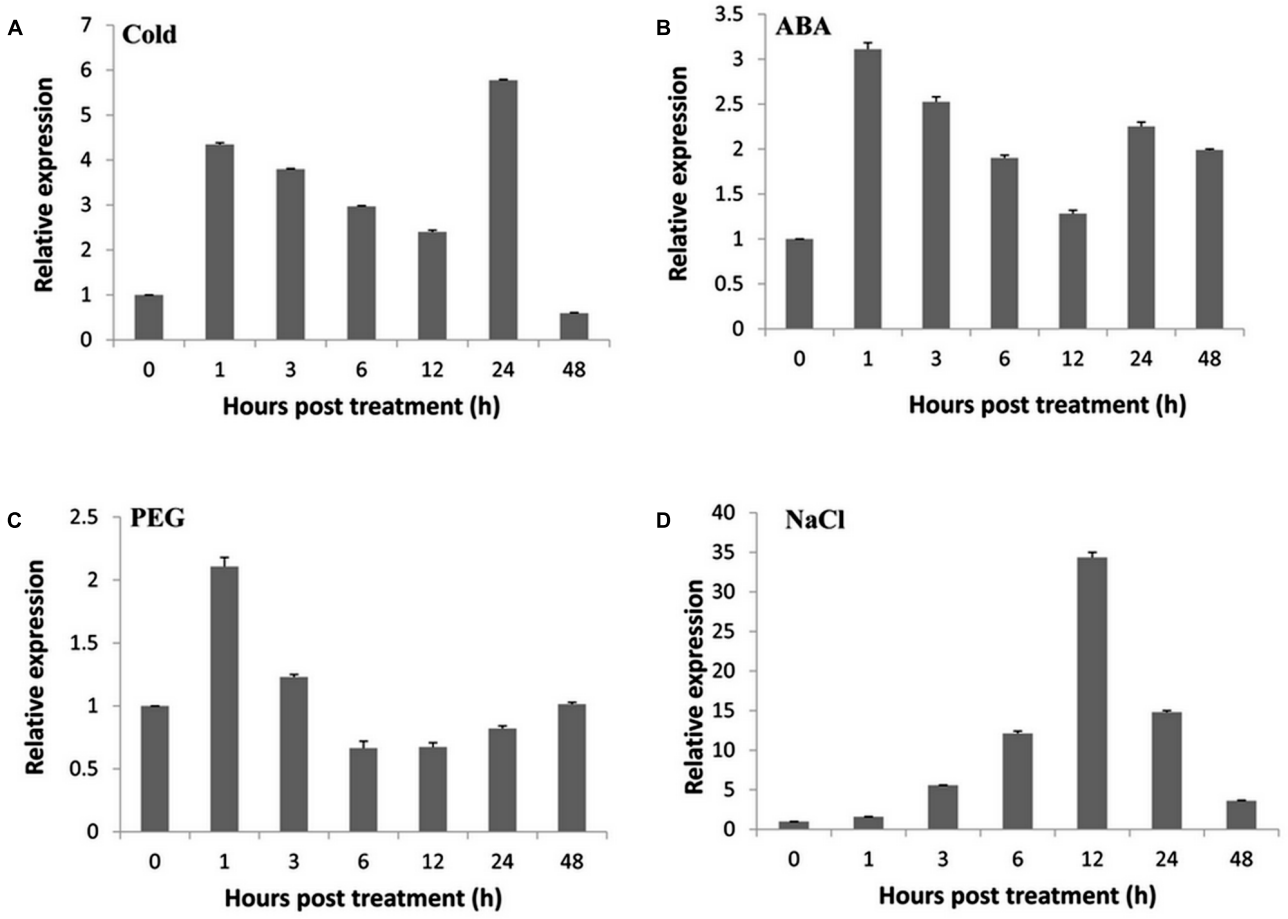
**Expression of the *TaNAC47* gene in wheat. (A)** Low-temperature (4°C) treatment; **(B)** 200 μM ABA treatment; **(C)** 16.1% PEG6000 treatment; **(D)** 250 mM NaCl treatment. The *y*-axis indicates the relative expression levels of the *TaNAC47* gene. The data were calculated using the 2^-ΔΔCT^ method. The experiment was performed three times. The bars show the standard errors.

To elucidate the molecular mechanism underlying the responsiveness to various abiotic stresses, RT-qPCR was performed to investigate the expression patterns of *TaNAC47* in different wheat tissues. The *TaNAC47* gene transcripts accumulated at the highest level in leaf but at low levels in young spike, stem and root (Supplementary Figure S1). Under cold stress conditions, *TaNAC47* expression was increased between 1 and 24 h (**Figure 1A**). Expression of *TaNAC47* was induced during the course of 1-48 h in the presence of exogenous ABA (**Figure 1B**). An increase in the *TaNAC47* transcript level was observed within 1 h of PEG-induced osmotic stress (**Figure 1C**). *TaNAC47* expression levels were also increased in response to NaCl treatment within 12 h, although the transcript level returned to background level afterwards (**Figure 1D**). These results indicated that gene expression for *TaNAC47* was significantly induced by all four abiotic stress treatments, and that *TaNAC47* was sensitive to cold, ABA and PEG stresses at an early stage of the treatments.

### *TaNAC47* Encoded Protein Localizes in the Nucleus and Acts as a Transcriptional Activator

**FIGURE 2:**
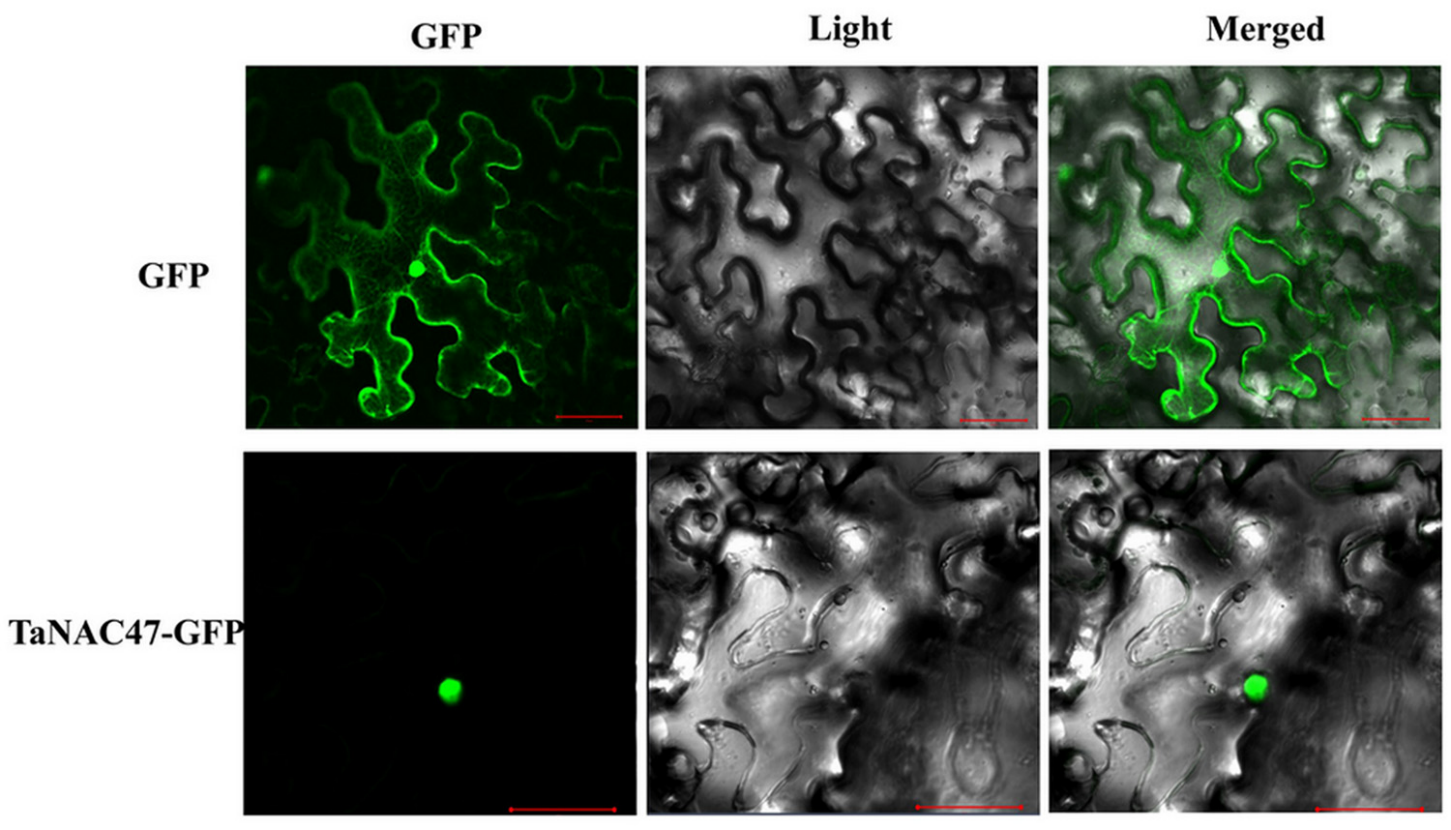
**Subcellular localization of the TaNAC47 protein in leaf epidermal cells of *Nicotiana benthamiana***. The p35:TaNAC47-GFP and p35:GFP constructs were separately introduced and expressed instantly in leaf epidermal cells of *Nicotiana benthamiana*. Bars = 30 μM.

To determine the subcellular localization of *TaNAC47* encoded protein, the full-length sequence of *TaNAC47* was fused to the GFP gene sequence in a pEarleyGate-GFP vector. Recombinant plasmids and pEarleyGate-GFP vector alone were transient expressed in the *N. benthamiana* leaves separately. Results obtained from confocal microscopy showed that the fluorescence signals from GFP alone were widely distributed throughout the cells, while the green fluorescent signals from transformed cells harboring *TaNAC47-GFP* were mainly observed in the nuclei (**Figure 2**). These results implied that the TaNAC47 protein was a nuclear-localized protein.

**FIGURE 3:**
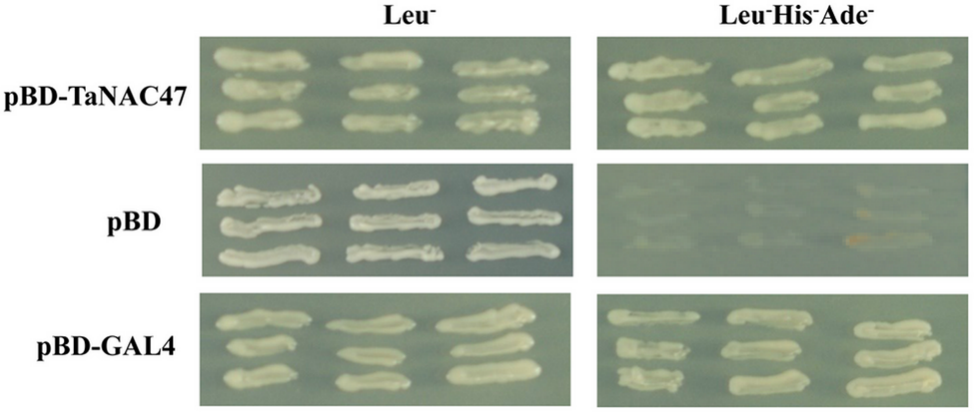
**Transactivation assay of the TaNAC47 protein in yeast**. Full-length TaNAC47 was fused with pDEST32. The pDEST32 vector alone and GAL4 were used as the negative and positive controls, respectively.

The transactivation assay in yeast showed that all transformants grew well on SD/Leu^-^ medium. While the transformants containing the *pDEST32* vector alone did not grow on the transactivation selective SD/Leu^-^His^-^Ade^-^ medium, the pDEST32 transformants fused with the ORFs of *TaNAC47* and pGAL4 grew on transactivation selective SD/Leu^-^His^-^Ade^-^ medium (**Figure 3**), demonstrating that these transformants were able to activate the transcription of the reporter genes *Ade* and *His* in the yeast strain AH109. Therefore, our results provided direct evidence that the TaNAC47 protein has transcriptional activity in yeast.

### Binding of TaNAC47 to the ABRE *Cis*-acting Element

**FIGURE 4:**
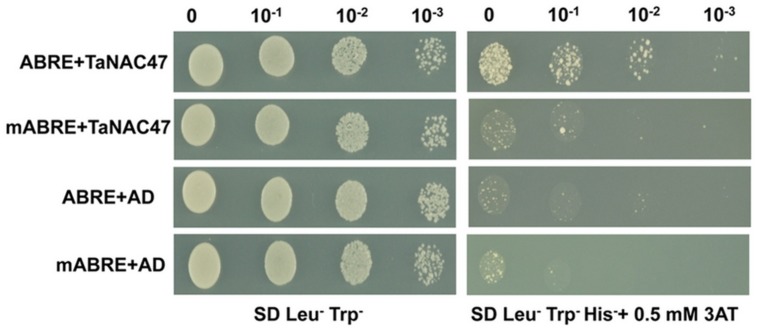
**The binding activity of TaNAC47 to ABRE element in a yeast one-hybrid system**. The hexamer ABRE/mABRE sequence was used as the negative control. Yeast cells grown in liquid medium were diluted in a 10× dilution series. A total of 10 μL of each dilution was spotted onto SD/Leu^-^Trp^-^His^-^ medium with 0.5 mM 3-AT.

The ABRE is an important *cis*-acting element for genes involved in the ABA signaling pathway. To investigate whether TaNAC47 participated in the ABA regulatory pathway, the binding affinity between TaNAC47 and ABRE was assessed by employing an yeast one-hybrid assay. As shown in **Figure 4**, all the yeast transformants were able to grow on the medium containing SD/Leu^-^Trp^-^. When adding 0.5 mM 3-aminotriazole (3-AT) to the medium, only the transformants containing the construct of *GAL4*-*AD*-*TaNAC47* and the ABRE bait plasmid were able to grow, but the control transformants were not. Therefore, our results strongly suggested that TaNAC47 had a capability to bind to ABRE.

### Overexpression of *TaNAC47* in *Arabidopsis* Increases Tolerance to Multiple Abiotic Stresses and Plant Sensitivity to ABA

**FIGURE 5:**
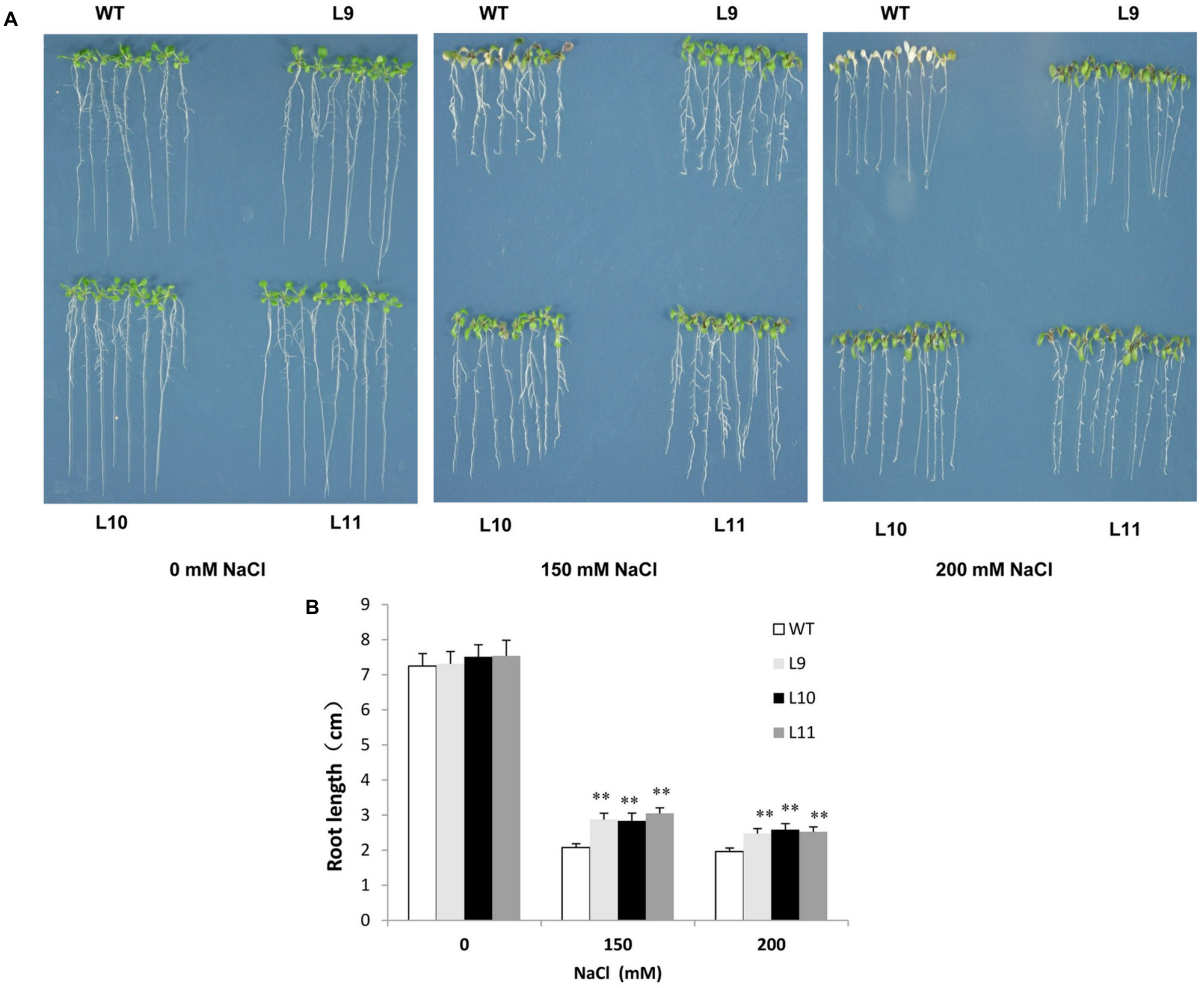
**Analysis of the enhanced salt tolerance in *TaNAC47*-overexpressing *Arabidopsis* seedling lines. (A)** Phenotype of WT and transgenic plants on MS with 0, 150, and 200 mM NaCl. **(B)** Root lengths of plants in **(A)**. The data represent the means from three experiments. The bars show the SD. Significant differences between the transgenic and WT lines are indicated as ^∗^0.01 < *P* < 0.05 and ^∗∗^*P* < 0.01.

To explore the function of *TaNAC47* in providing tolerance to abiotic stress in plants, transgenic *Arabidopsis* plants over-expressing *TaNAC47* driven by the *CaMV* 35S promoter were generated. At least 15 transgenic lines were acquired, and three independent homozygous lines with relatively high expression levels (Supplementary Figure S2) were used for the analysis. For salt tolerance, no obvious difference was observed between WT and transgenic plants under normal conditions. However, leaf growth in WT plants was delayed, their cotyledons turned yellow and some of the cotyledons exhibited albino characteristics in the presence of 150 and 200 mM NaCl. Additionally, root growth remained healthier in transgenic plants than that in WT plants during the time period when raising the NaCl concentration to 150 and 200 mM (**Figures 5A,B**).

**FIGURE 6:**
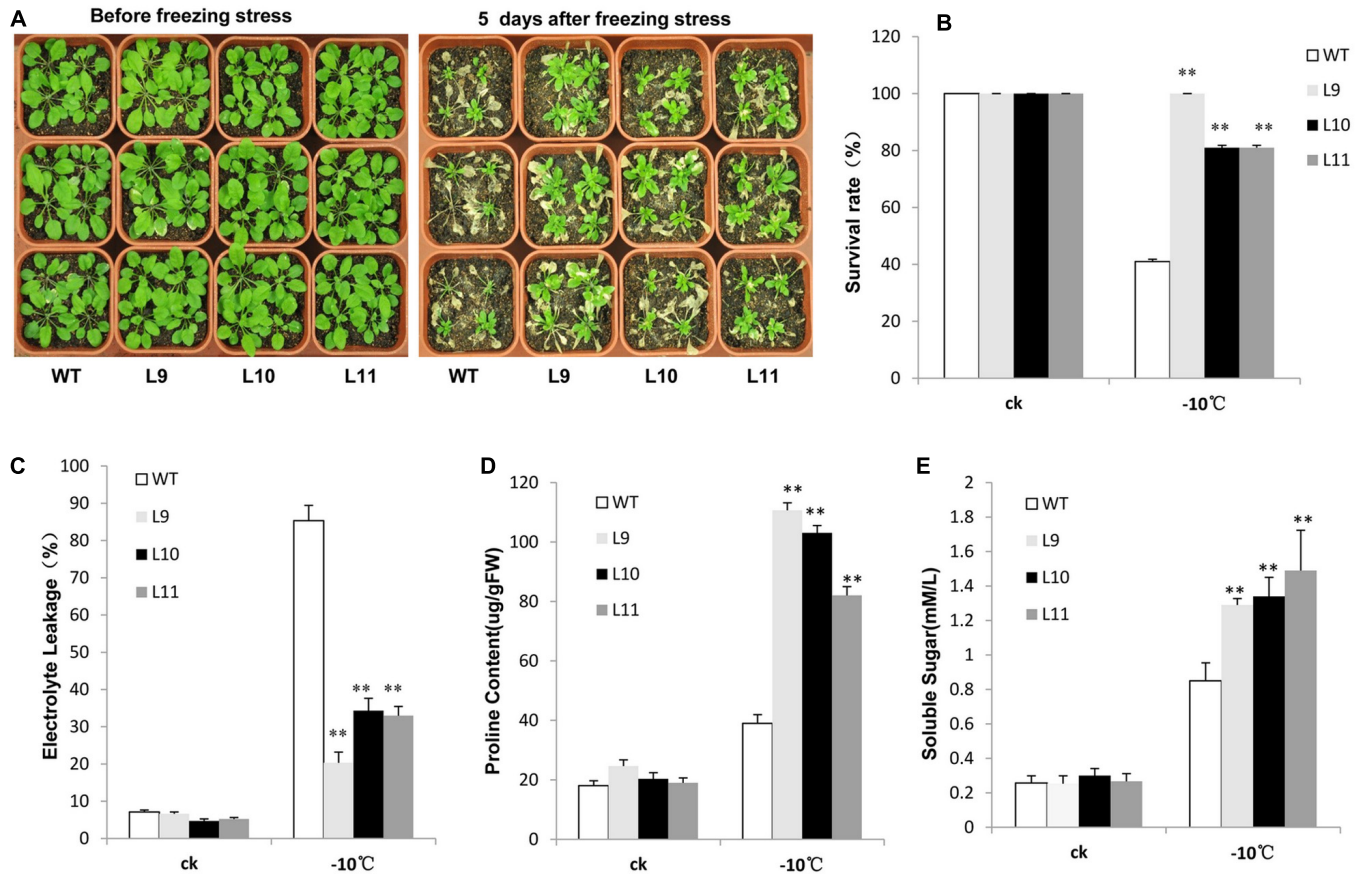
**Analysis of the enhanced freezing tolerance in *TaNAC47*-overexpressing *Arabidopsis* seedling lines. (A)** Phenotype of WT and transgenic plants before and after freezing treatments. **(B)** Survival rate after freezing. **(C)** Relative electrolyte leakage. **(D)** Proline content. **(E)** Soluble sugar content. The data represent the means from three experiments. The bars show the SD. Significant differences between the transgenic and WT lines are indicated as ^∗^0.01 < *P* < 0.05 and ^∗∗^*P* < 0.01.

For freezing tolerance, 2-week-old plants were exposed to -10 °C for 3 h and then transferred to normal 22°C growing conditions for recovery. As a result, over half of WT plants were dead with a survival rate at approximately 41%. In contrast, the survival rate for transgenic plants was observed at 81–100% (**Figures 6A,B**). Additionally, the electrolyte leakage was lower in transgenic plants relative to WT plants when they were at the freezing condition (**Figure 6C**). Transgenic plants further produced remarkably high levels of proline and soluble sugars under freezing condition compared to WT plants (**Figures 6D,E**).

**FIGURE 7:**
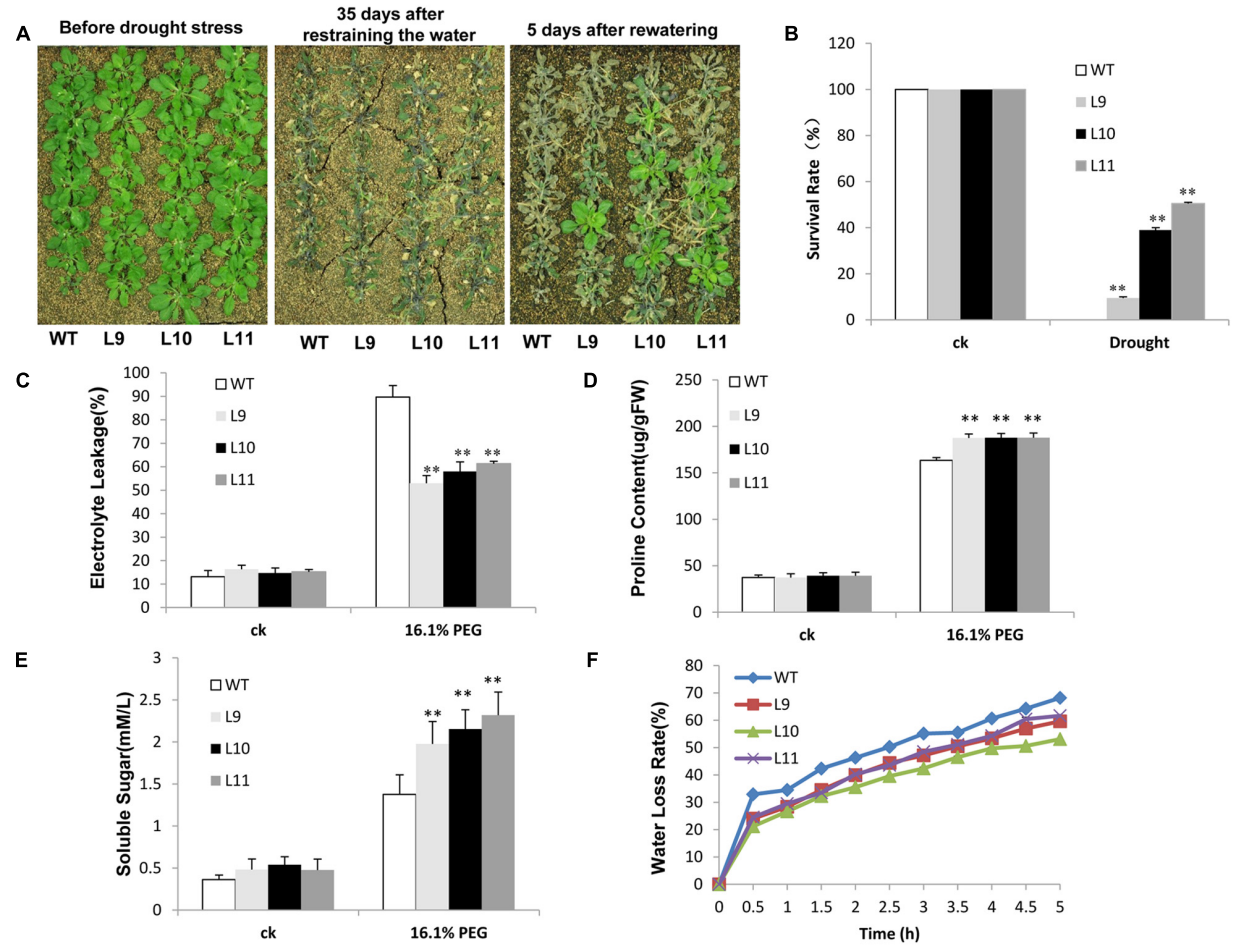
**Analysis of the enhanced drought tolerance in *TaNAC47*-overexpressing *Arabidopsis* seedling lines. (A)** Phenotype of WT and transgenic plants before and after drought treatments. **(B)** Survival rate after drought treatment. **(C)** Relative electrolyte leakage. **(D)** Proline content. **(E)** Soluble sugar content. **(F)** Water loss rate of the detached leaves. The data represent the means from three experiments. The bars show the SD. Significant differences between the transgenic and WT lines are indicated as ^∗^0.01 < *P* < 0.05 and ^∗∗^*P* < 0.01.

The drought tolerance study was evaluated in soils. Soon after irrigation was suspended, no discernible developmental differences were observed between transgenic and WT plants. However, after withholding water for 35 days, WT plants showed visible symptoms of drought-induced damage, such as drying, wilting, and even death. In contrast, some transgenic plants remained green with expanded leaves. Further analyses showed that after re-watering the survival rate was significantly different between transgenic and WT plants. Few WT plants survived, whereas about 10–51% of transgenic plants continued to grow (**Figures 7A,B**). When plants were grown under drought stress condition, the electrolyte leakage was lower in transgenic plants than in WT plants (**Figure 7C**). Transgenic plants further showed remarkably higher levels of proline and soluble sugars relative to those found in WT plants (**Figures 7D,E**). The water-loss rates were also lower in transgenic plants than in WT plants (**Figure 7F**).

**FIGURE 8:**
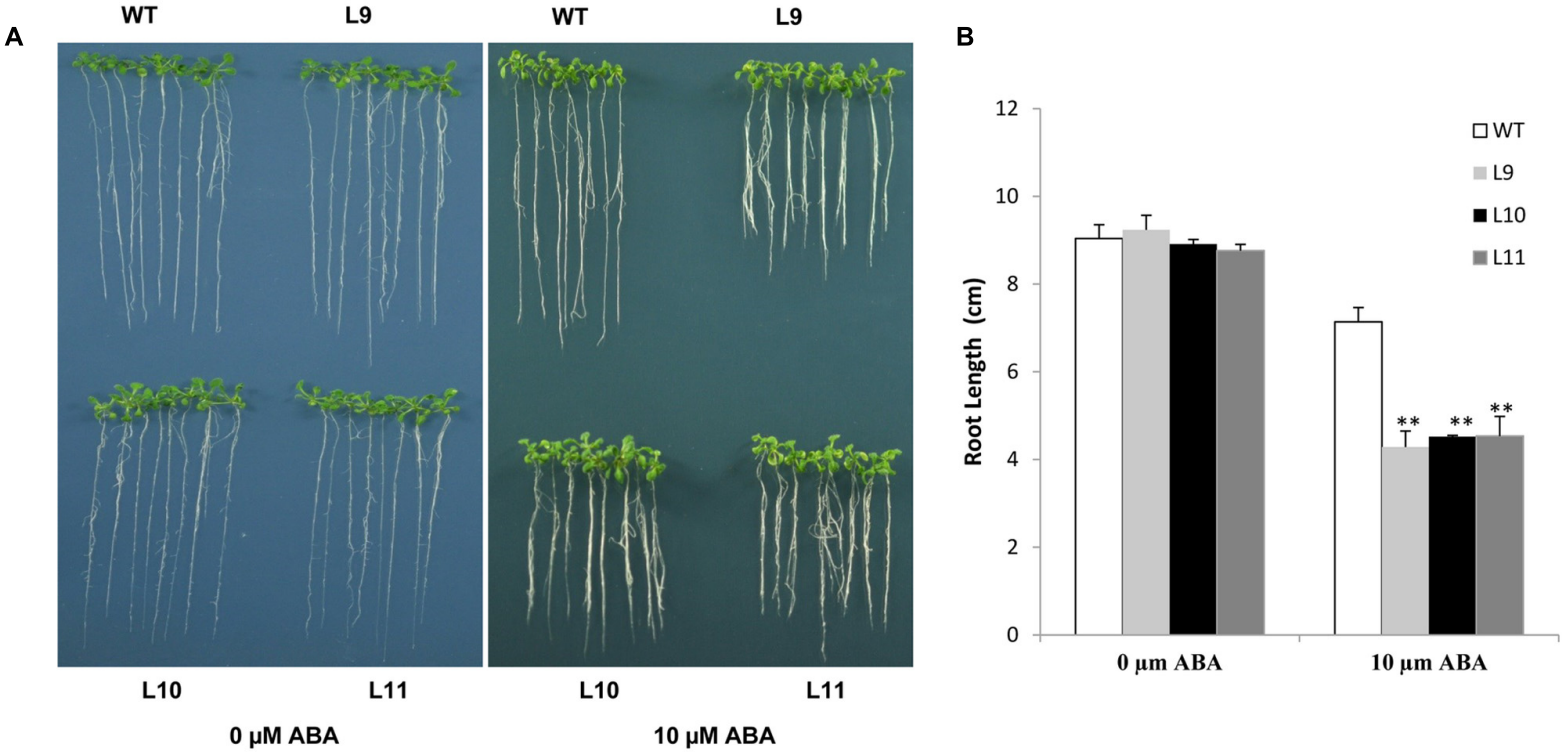
**Responses of *TaNAC47*-overexpressing transgenic plants to ABA. (A)** Phenotype of the transgenic and WT plants on MS with 0 and 10 μM ABA. **(B)** Root lengths of plants on MS with 10 μM ABA. The data represent the means from three experiments. The bars show the SD. Significant differences between the transgenic and WT lines are indicated as ^∗^0.01 < *P* < 0.05 and ^∗∗^*P* < 0.01.

The ABA sensitivity in transgenic plants was assessed. In the absence of ABA, no differences in root length were found between transgenic and WT plants under normal growing conditions. However, the root growth was significantly inhibited in transgenic plants, but not in WT plants in the presence of 10 μM ABA (**Figure 8**). These results suggested that overexpression of the *TaNAC47* gene led to increased sensitivity to ABA, which resulted in retarded growth of transgenic plants.

### Altered Expression of Stress-Responsive Genes in Transgenic *TaNAC47* Plants

**FIGURE 9:**
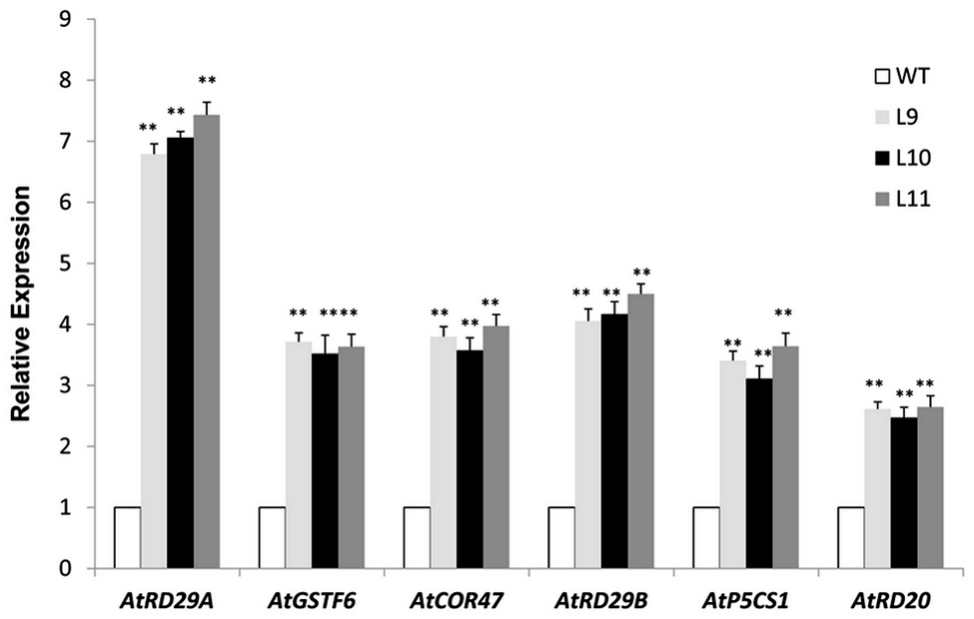
**Expression levels of the stress-associated genes under salt stress**. Gene-specific primers were used to detect the relative transcript levels of the stress-related genes. The data represent the means from three experiments. The bars show the SD. Significant differences between the transgenic and WT lines are indicated as ^∗^0.01 < *P* < 0.05 and ^∗∗^*P* < 0.01.

The *TaNAC47* transgenic plants exhibited an improved tolerance to freezing, drought and salt stresses. Next, we examined whether expressions of any other genes involving stress response were also altered in these transgenic plants. When the plants were stressed from the salt treatment, the expression levels of genes *AtRD29A*, *AtRD29B*, *AtCOR47*, *AtRD20*, *AtGSTF6*, and *AtP5CS1* were increased in *TaNAC47* transgenic plants compared to WT plants (**Figure 9**). We searched the *Arabidopsis* genome database to acquire the promoter regions of these up-regulated genes. Results showed that these genes also contained the NAC core motif CGT[G/A] within their promoter regions (Jiang G et al., 2014), implying that *TaNAC47* may confer stress tolerance through regulating various stress-responsive genes.

## Discussion

The NAC superfamily is one of the largest TF families found only in plants (Wang N et al., 2013b). We characterized wheat *TaNAC47* gene, a novel stress-related member of the NAC gene family in wheat. High protein sequence identity was found between TaNAC47 and another reported stress responsive NAC protein TaNAC67. Although they shared high sequence identity, these two genes showed several intrinsic differences. First is the gene structure difference, no intron was detected in the *TaNAC47* gene, while the *TaNAC67* contained one intron and two exons. Secondly, a significant difference was observed in their expression levels and response times (Mao et al., 2014). Finally, although overexpression of the two genes both enhanced the tolerance to cold, drought and salt stresses in transgenic plants, only the seedlings overexpressing the *TaNAC47* gene increased the sensitivity to ABA (**Figure 8**). The promoter region of *TaNAC47* contained not only ABRE but also A-box, C-box and W-box elements, which were known to participate in abscisic acid responsiveness (Supplementary Table S1), the presence of these *cis*-elements may explain why *TaNAC47* was induced more quickly in response to ABA treatment compared with *TaNAC67*.

We found tolerances to salt, cold, and drought stresses were likely conferred by overexpressing *TaNAC47* in transgenic plants. This was supported by the results from stress-induced expression profiles of the *TaNAC47* gene. There is a strong correlation between stress induction of the gene and stress tolerance expressed in the corresponding transgenic plants based on the physiological and molecular changes in the plants. At the physiological and biochemical levels, soluble sugars, and proline contents in plants overexpressing *TaNAC47* have increased after drought and cold treatments. This agreed with previous report showing the increase in proline under stress in plants was associated with stress tolerance (Wang C et al., 2013). Furthermore, proline contributed to the adjustment of osmotic balance and induced the expression of stress-related genes (Ashraf and Foolad, 2007). The enhancement in soluble sugar content was thought to relate to protecting cellular functions, such as maintaining the structure of cellular components or acting as signal molecules (Seki et al., 2007). In addition to these physiological changes, the rate of leaf water-loss and the survival rate were both used as typical physiological indices to investigate plant resistance. Plants showing higher survival rates and less water-loss had higher tolerance and were more resistant to stresses. In this study, the leaf water-loss and the survival rate of the *TaNAC47* transgenic plants were consistent with previous reports from the transcription factors OsNAC52 and RhNAC3 (Gao et al., 2010; Jiang X et al., 2014). The changes in the physiological indices in *TaNAC47* transgenic plants likely resulted in enhancing tolerance to adverse conditions at the physiological level.

At gene transcription level, transgenic plants overexpressing *TaNAC47* exhibited up-regulation of various other stress responsive genes, including *AtRD29A, AtRD29B, AtCOR47, AtRD20, AtGSTF6*, and *AtP5CS1* (**Figure 9**). Putative NAC-binding *cis*-elements were found in the promoter sequences of these six genes, suggesting that these genes might be transcriptionally regulated directly by *TaNAC47* (Jiang G et al., 2014). Furthermore, the RhNAC3 and AhNAC2 transcription factors were shown to enhance the expression of these genes and bind to the putative NAC recognition site of the *AtRD29A*, *AtCOR47* and *AtRD20* genes based on EMSA (Liu et al., 2011; Jiang G et al., 2014). The *P5CS1* gene was involved in the proline biosynthetic pathway and was induced under stress conditions (Ashraf and Foolad, 2007), thereby contributing to the accumulation of proline contents in transgenic plants and making plants more resistant to stress. GSTF6 was found to play vital roles in plant stress tolerance and detoxification (Lan et al., 2013). Most of the up-regulated genes in *TaNAC47* transgenic plants were likely involved in the ABA-dependent signaling pathway. We showed overexpression of *TaNAC47* resulted in enhanced ABA sensitivity, which was consistent with previous studies on the RhNAC3, SNAC2 and OsNAC52 transcription factors from other species (Hu et al., 2008; Gao et al., 2010). ABRE is regarded as the major type of *cis*-element involved in the ABA signaling pathway. Previous studies on the interaction of other NAC transcription factors with ABRE were not available. In this study, we demonstrated the binding of the TaNAC47 protein to ABRE based on a yeast one-hybrid assay. Thus, our results strongly implied that TaNAC47 may participate in the ABA-dependent signaling pathway.

## Conclusion

The functions of a novel wheat *TaNAC47* gene were evaluated in this study overexpression of *TaNAC47* in *Arabidopsis*, in general, enhanced the tolerance of transgenic plants to drought, freezing and salt stresses, and led to increased ABA sensitivity. Furthermore, an interaction between *TaNAC47* encoded protein and ABRE *cis*-element was also revealed. Our data indicated that *TaNAC47* regulates plant adaptation to various abiotic stresses and is a potential candidate gene to improve stress tolerance in crops.

## Author Contributions

LNZ, LCZ, CX and GYZ performed most of the experiments. LNZ, LCZ, JZJ and XYK designed the experiments and wrote the paper. All authors read and approved the final manuscript.

## Conflict of Interest Statement

The authors declare that the research was conducted in the absence of any commercial or financial relationships that could be construed as a potential conflict of interest.

## Footnotes

1 http://expasy.org/tools/protparam.html.

2 http://www.expasy.ch/tools/scanprosite/.

3 http://www.dna.affrc.go.jp/PLACE/.
