# Supplementary material for: The Novel Wheat Transcription Factor TaNAC47 Enhances Multiple Abiotic Stress Tolerances in Transgenic Plants
Source: Front Plant Sci. 2016 Jan 18;6:1174. doi: 10.3389/fpls.2015.01174 (PMC4716647; doi:10.3389/fpls.2015.01174)
Supplement: Supplementary file 1 [file Table_1.PDF]

**Supplementary Material**

Table 1. The *cis*-acting elements identified in the promoter regions of *TaNAC47*

| Name | Number | Sequence function |
|---|---|---|
| ABRE | 6 | *cis*-acting element involved in the abscisic acid responsiveness |
| A-box | 1 | Plant bZIP protein DNA binding sequence with an ACGT core *cis*-element |
| C-box | 1 | Plant bZIP protein DNA binding sequence with an ACGT core *cis*-element |
| CAAT-box | 10 | common cis-acting element in promoter and enhancer regions |
| DRE/CRT | 2 | cis-acting element involved in drought, high-salt and cold responsiveness |
| GATA-box | 11 | common cis-acting element in promoter regions |
| I-box | 4 | cis-acting element involved in light responsiveness |
| HSE | 3 | cis-acting element involved in heat stress responsiveness |
| LTRE | 5 | cis-acting element involved in low-temperature responsiveness |
| T/G-box | 3 | cis-acting element involved in jasmonate signaling responsiveness |
| W- box | 7 | WRKY protein recognition site involved in abscisic acid responsiveness |
| MYBRS | 8 | MYB protein recognition site involved in dehydration responsiveness |
| MYCRS | 3 | MYC protein recognition site involved in dehydration responsiveness |

The *cis*-elements were identified in the upstream of promoters (1500 bp)
