# Supplementary material for: The Novel Wheat Transcription Factor TaNAC47 Enhances Multiple Abiotic Stress Tolerances in Transgenic Plants
Source: Front Plant Sci. 2016 Jan 18;6:1174. doi: 10.3389/fpls.2015.01174 (PMC4716647; doi:10.3389/fpls.2015.01174)
Supplement: Supplementary file 2 [file Table_2.PDF]

Supplementary Material

Table 2.The gene-specific primers selected for quantitative RT-PCR

Gene name	Accession	Forward primers(5'-3')	Reverse primers(5'-3')
TaTubulin	AF251217	TTAGACTTGCGAAGCCAGCA	AAATGCCCTTGAGGTTTCCC
AtActin	NM_112764	CTCATGCCATCCTCCGTCTT	ACTTGCCCATCGGGTAATTC
AtRD29A	NM_124610	GAGCAACGAGGGGAAGATAAAAG	TCAGTCGCACCACCACCGAACCA
AtRD29B	NM_124609	CAAAACCAAGCACCTACACA	CTCCTTCACTCCACTTCCAC
AtP5CS1	AB022784	GCAGAGCTATTCCTTCGC	ATTCCATTGTCTCCGTCG
AtRD20	NM_128898	ATTCGAGCACCTATGACACC	AAACTTCCATCAAAGCAACC
AtCOR47	NM_101894	TCCCAGGACACCACGACAAGAC	CCTCTTCAGTGGTCTTGGCATG
AtGSTF6	NM_100174	ACTCTTCGACGAGCGTCCACAT	GGTCATCGCCACTTTTATTACA
