# Supplementary figures and images for: The Novel Wheat Transcription Factor TaNAC47 Enhances Multiple Abiotic Stress Tolerances in Transgenic Plants
Source: Front Plant Sci. 2016 Jan 18;6:1174. doi: 10.3389/fpls.2015.01174 (PMC4716647; doi:10.3389/fpls.2015.01174)

**Figure S1**

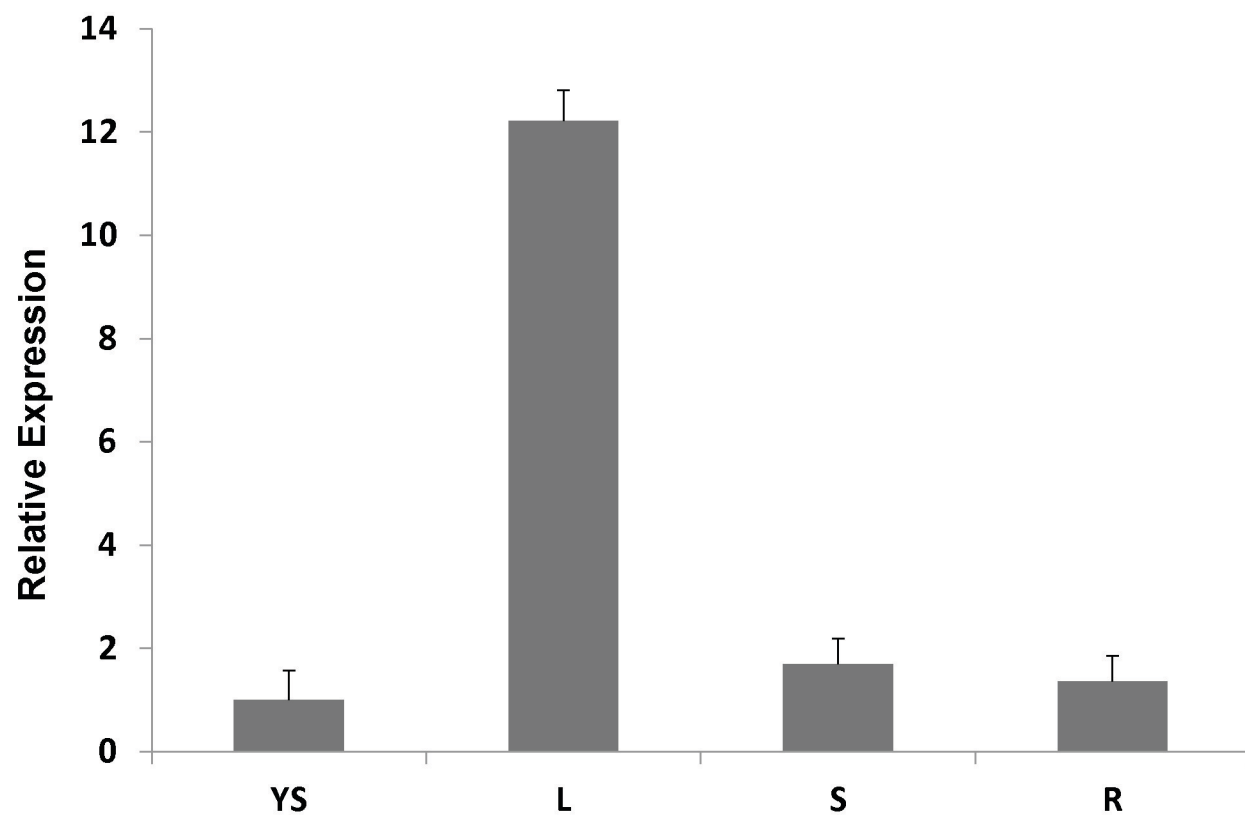

Supplement: Supplementary file 3 [file Image_1.PDF]

**Figure S2**

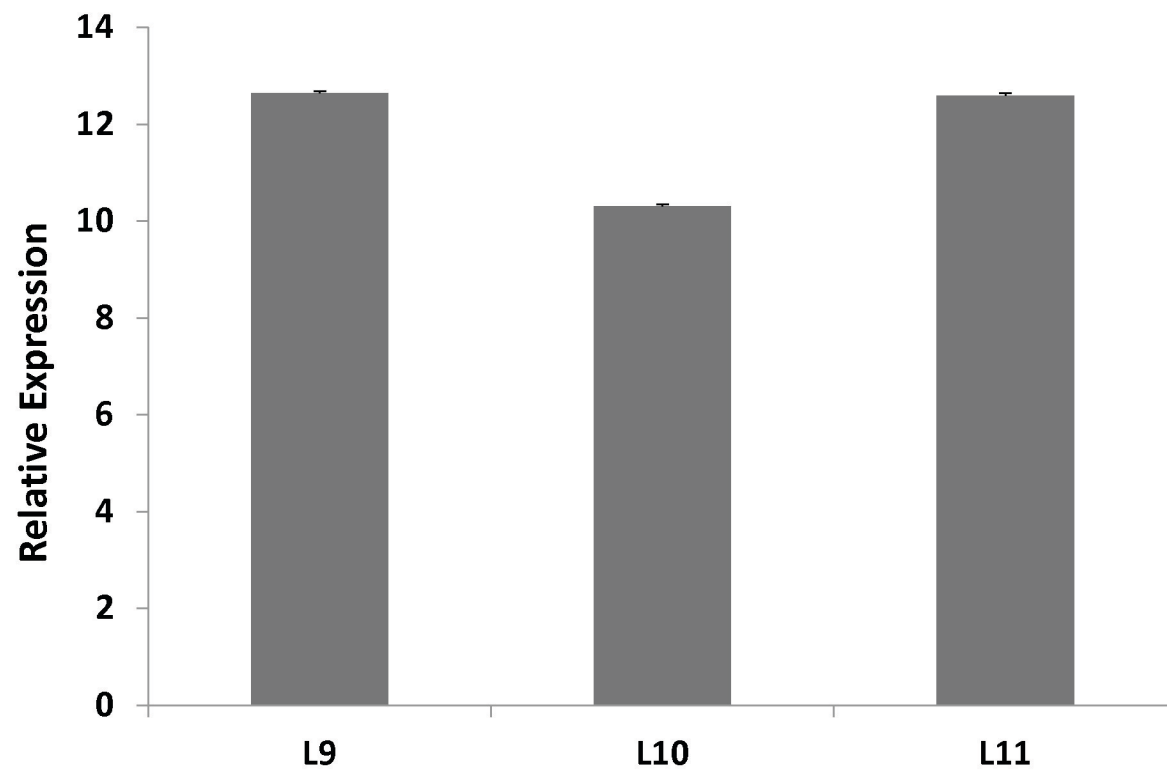

Supplement: Supplementary file 4 [file Image_2.PDF]
